# Hyperandrogenism, Elevated 17-Hydroxyprogesterone and Its Urinary Metabolites in a Young Woman with Ovarian Steroid Cell Tumor, Not Otherwise Specified: Case Report and Review of the Literature

**DOI:** 10.1155/2019/9237459

**Published:** 2019-10-27

**Authors:** Felix C. K. Wong, Angela Z. Chan, W. S. Wong, Angel H. W. Kwan, Tracy S. M. Law, Jacqueline P. W. Chung, Jeffrey S. S. Kwok, Angel O. K. Chan

**Affiliations:** ^1^Department of Chemical Pathology, Prince of Wales Hospital, The Chinese University of Hong Kong, Shatin, Hong Kong; ^2^Department of Anatomical and Cellular Pathology, Prince of Wales Hospital, The Chinese University of Hong Kong, Shatin, Hong Kong; ^3^Department of Medicine, North District Hospital, Sheung Shui, Hong Kong; ^4^Department of Obstetrics and Gynaecology, Prince of Wales Hospital, The Chinese University of Hong Kong, Shatin, Hong Kong

## Abstract

We describe a case of a 24-year-old overweight woman who presented with hirsutism, secondary amenorrhea, clitoromegaly, and symptoms of diabetes mellitus (DM). While a diagnosis of polycystic ovary syndrome (PCOS) with its associated metabolic disturbances was initially considered, serum total testosterone, androstenedione, and 17-hydroxyprogesterone (17-OHP) measured by liquid chromatography tandem mass spectrometry (LC-MS/MS) were significantly increased. As 17-OHP did not increase upon ACTH (Synacthen) stimulation and the urinary steroid profile (USP) was compatible with an ovarian source of 17-OHP excess rather than adrenal, non classical congenital adrenal hyperplasia (NCCAH) was unlikely and an androgen-secreting tumor was suspected. Transabdominal ultrasound revealed the presence of an enlarged right ovary with a polycystic ovary morphology and no discrete mass. Transvaginal ultrasound and [^18^F]− fluorodeoxyglucose positron emission tomography–computed tomography (FDG PET–CT) enabled the localization of a right ovarian tumor. Laparoscopic right salpingo-oophorectomy was performed and a histological diagnosis of steroid cell tumor, not otherwise specified (SCT–NOS) was made. Hyperandrogenism and menstrual disturbances resolved postoperatively. A literature review revealed that 17-OHP-secreting SCT–NOS may uncommonly show positive responses to ACTH stimulation similar to 21-hydroxylase deficiency. Alternatively, USP might be useful in localizing the source of 17-OHP to the ovaries. Its diagnostic performance should be evaluated in further studies.

## 1. Introduction

Identifying the underlying cause and localizing the source of elevated androgens or their precursors in woman with hyperandrogenism can be challenging. In the setting of female hyperandrogenism, a significant elevation of 17-hydroxyprogesterone (17-OHP) is often suggestive of a diagnosis of congenital adrenal hyperplasia (CAH), most commonly 21-hydroxylase deficiency. Nevertheless, androgen-secreting tumors, which may be of adrenal or ovarian origin, may also be responsible for such finding. Investigations into an increased 17-OHP generally include serum androgen profile, ACTH stimulation test, urinary steroid profile (USP), and if biochemically compatible with 21-hydroxylase deficiency, confirmation by molecular testing of the CYP21A2 gene. Here, we present the diagnostic challenges in a young woman with hirsutism and significantly elevated total testosterone, androstenedione, and 17-OHP in whom the diagnosis of a hormone-secreting steroid cell tumor, not otherwise specified (SCT–NOS), was made.

## 2. Case Presentation

**Figure 1:**
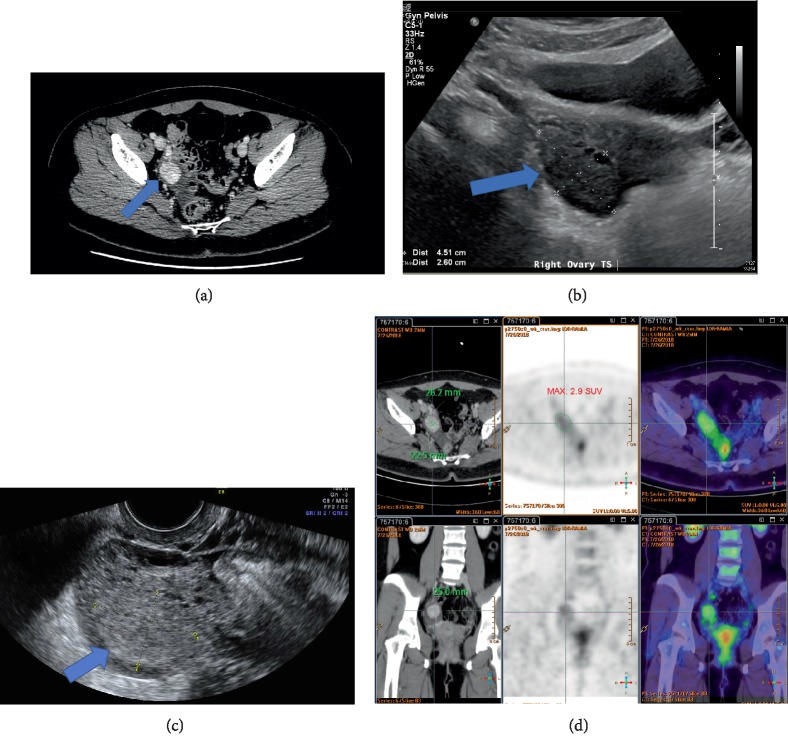
(a) Contrast CT scan of the abdomen and pelvis showed an enlarged 2.6 × 2.3 × 2.5 cm right ovary (blue arrow). (b) Transabdominal ultrasound showed an enlarged 4.5 × 2.6 × 4.5 cm right ovary five months after CT scan (blue arrow) (c) Transvaginal ultrasound showed a 2.6 × 2.4 × 2.2 cm hyperechoic solid tumor inside the right ovary (blue arrow) (d) FDG PET–CT scan with contrast showed a 2.6 × 2.3 × 2.5 cm mildly FDG-avid mass (SUV_max_ 2.9 g/ml) in the right ovary (tumor dimensions labelled).

A 24-year-old woman was referred to the gynecology clinic for a one-year history of secondary amenorrhea and symptoms of hyperandrogenism (acne and hirsutism) for two years. She had oligomenorrhea for one year prior to developing amenorrhea. She enjoyed good past health except for a referral for pediatric assessment for overweight [Body mass index (BMI) 24.9] at the age of 12 with no specific underlying cause identified. Menarche occurred at the age of 12 years. Examination revealed that she is overweight (BMI 26.0) with normal blood pressure. Acne was present but there was no obvious hirsutism (hair removal was done for cosmetic reasons). Other clinical features of Cushing's syndrome were absent. Pregnancy test was negative. Transabdominal ultrasound showed an enlarged (16.6 cm^3^) right ovary with more than 12 small follicles (< 9 mm), which represents sonographic evidence of polycystic ovary based on the Rotterdam criteria [1]. The left ovary was normal in appearance and size (6.8 cm^3^). Polycystic ovary syndrome (PCOS) was suspected and combined oral contraceptive pills were prescribed with a return of menstruation. Further laboratory testing, however, demonstrated a significant elevation of serum total testosterone (10.6 nmol/L), androstenedione (28.2 nmol/L), and 17-hydroxyprogesterone (17-OHP) (52 nmol/L) all measured by liquid chromatography tandem mass spectrometry (LC-MS/MS). 1 mg overnight dexamethasone suppression test was negative for Cushing's syndrome (Table 1). In view of the grossly elevated androgens and 17-OHP, PCOS was unlikely. In addition, the raised 17-OHP raised the possibility of non classical congenital adrenal hyperplasia (NCCAH). On further questioning and physical examination, the patient noticed deepening of her voice in the recent two years, and mild clitoromegaly was found. Furthermore, the patient complained of polyuria and polydipsia for 6 months and a diagnosis of diabetes mellitus was confirmed by laboratory tests (fasting glucose 7.2 mmol/L, HbA1c 6.9%). Metformin 250 mg twice daily was prescribed. 250 *μ*g ACTH (Synacthen) stimulation test was performed to exclude NCCAH. While the baseline and stimulated 17-OHP concentrations were both compatible with NCCAH due to 21-hydroxylase deficiency (> 30 nmol/L based on the endocrine society clinical practice guideline [2]), a flat response was noted (Table 2), which was atypical of NCCAH. A 24-h urinary steroid profile (USP) by gas chromatography mass spectrometry (GC–MS) revealed moderate elevation of androgen metabolites (androsterone: 5855 *μ*g/day, reference interval: 431–2037 *μ*g/day; etiocholanolone: 2175 *μ*g/day, reference interval: 198–1551 *μ*g/day), and gross elevation of two of the three metabolites of 17-OHP (17-hydroxypregnanolone: 3358 *μ*g/day, reference interval: 16–295 *μ*g/day; pregnanetriol: 5338 *μ*g/day, reference interval 132–1156 *μ*g/day). 11-oxopregnanetriol, which is another metabolite of 17-OHP, was however normal (7 *μ*g/day, reference interval: < 44 *μ*g/day). Cortisol and corticosterone metabolites were not in excess. The USP findings suggested an ovarian source of 17-OHP rather than adrenal. Contrast computed tomography (CT) of the abdomen and pelvis showed a 2.6 × 2.3 × 2.5 cm ovoid enhancing soft tissue over the right adnexal region, commented to be likely the right ovary, with a small local surrounding fluid collection (Figure 1(a)). No adrenal mass was identified. Transabdominal pelvic ultrasound performed five months later showed an enlarged right ovary measuring 4.5 × 2.6 × 4.5 cm in size with a volume of 27.6 cm^3^. There were more than 12 tiny follicles with sizes measuring up to 8 mm present at the periphery of the right ovary, representing sonographic evidence of polycystic ovary. The left ovary measured 2.6 × 2.2 × 2.0 cm in size with a volume of 5.8 cm^3^. Similar to the CT scan obtained earlier, no obvious mass was found in either ovaries (Figure 1(b)). As NCCAH was less likely and severe hyperandrogenism was present, further imaging was arranged for the localization of an androgen-secreting tumor. Transvaginal ultrasound of the pelvis revealed a 2.6 × 2.4 × 2.2 cm hyperechoic solid tumor inside the right ovary with hypervascularity seen on Doppler and multiple follicles at the periphery of the right ovary (Figure 1(c)). The left ovary also appeared to be polycystic in appearance. Whole body [^18^F]− fluorodeoxyglucose positron emission tomography–computed tomography (FDG PET–CT) scan with contrast showed a 2.6 × 2.3 × 2.5 cm mildly fluorodeoxyglucose–avid mass (SUV_max_ 2.9 g/ml) in the right ovary with homogeneous solid contrast enhancement and no calcification, fat or cystic component. There were no hypermetabolic lymph nodes seen in the abdomen and pelvis (Figure 1(d)). A diagnosis of an androgen-secreting tumor in the right ovary was made. Laparoscopic right salpingo-oophorectomy, diagnostic hysteroscopy and curettage of the uterus were performed. Peritoneal fluid was obtained for cytology. Intraoperatively, an enlarged right ovary measuring 4.5 × 4.5 cm was found. The left ovary had a polycystic ovary-like appearance with flimsy adhesions to the ovarian fossa and adhesiolysis was performed. The resected right ovary was cut open, revealing the presence of an orange-colored tumor inside.

**Figure 2:**
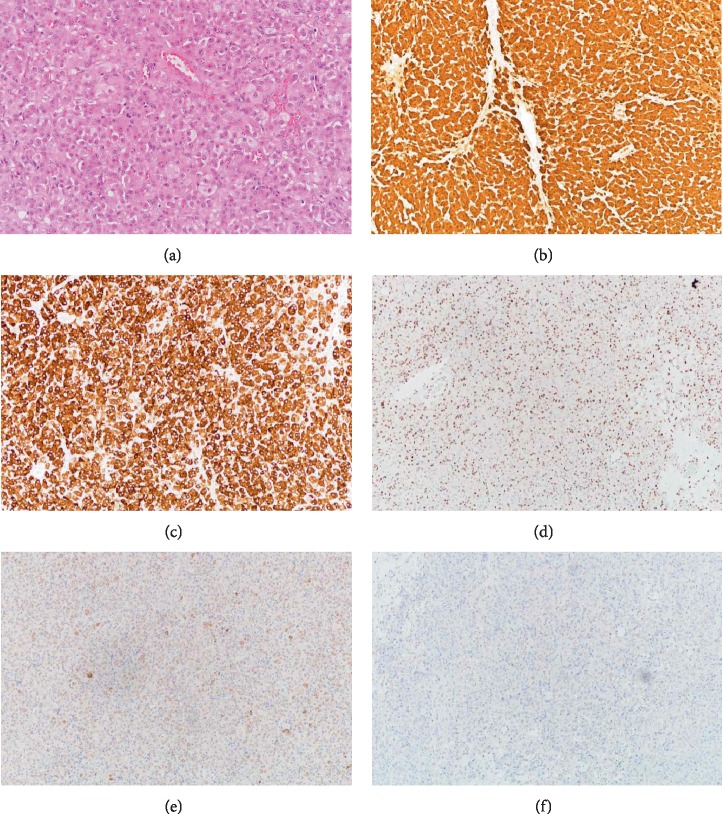
(a) Microscopic appearance of the tumor (20x magnification); (b) Calretinin immunostain (10x magnification): Positive; (c) Inhibin immunostain (10x magnification): Positive; (d) Androgen receptor immunostain (10x magnification): Positive; (e) Melan-A immunostain (10x magnification): Weakly positive; and (f) CD-99 (10x magnification) immunostain: Negative.

Microscopic examination of the specimen revealed polygonal cells arranged in diffuse sheets with round nuclei, prominent nucleoli, and abundant granular eosinophilic to pale vacuolated cytoplasm. Mitotic figures were inconspicuous (<1 per 10 high power fields). No other negative prognostic features of steroid cell tumors (i.e., necrosis, haemorrhage, and nuclear atypia) were found. No Reinke crystals were identified. Immunostaining was positive for inhibin, calretinin, and androgen receptor, weakly positive for Melan-A, and negative for CD99 (Figure 2). The diagnosis was steroid cell tumor, not otherwise specified (SCT–NOS). Uterine curettage did not yield sufficient material for analysis. Cytology of the peritoneal fluid was negative for malignant cells.

The patient recovered uneventfully after the operation, with a normalization of testosterone, androstenedione, and 17-OHP concentrations and attainment of a luteal phase progesterone concentration two weeks after the operation (Table 1). Postoperative HbA1c was 6.3–7.0%, while she continued metformin at the same dosage (HbA1c at diagnosis was 6.9%, see Table 1). No obvious improvement in her glycemic control was seen after the operation. The patient regained spontaneous menstruation within one month after the operation with improvement in acne and subsequent menstrual cycles were regular and 28 days each. A transvaginal ultrasound performed five months postoperatively showed a persistent polycystic ovary (PCO) morphology of the left ovary with 20 antral follicles and an ovarian volume of 10 cm^3^. She remained clinically well 9 months postoperatively.

## 3. Discussion

Androgen-secreting tumors are a rare cause of hyperandrogenism in women (0.2%) [3]. In contrast to PCOS in which the main presentation of hyperandrogenism is hirsutism, androgen-secreting tumors present with progressive and more severe hyperandrogenism leading to signs of virilization, e.g., clitoromegaly, deepening of voice, and male-pattern hair loss. Biochemically, these tumors are associated with total testosterone levels greater than 5.2 to 6.9 nmol/L [4–6]. Androgen-secreting tumors in females could be of adrenal or ovarian origin. While adrenal tumors are almost always detectable by CT scan [7, 8], ovarian androgen-secreting tumors may be small and difficult to be localized, often requiring a combination of imaging techniques and a high index of suspicion, and venous sampling was sometimes performed to locate the source of androgen secretion [5, 6, 9, 10]. In our case, both transabdominal ultrasound and CT scan showed an enlarged right ovary, but were not able to demonstrate the presence of a discrete tumor mass arising from the right ovary. Transvaginal ultrasound and FDG PET–CT scan were required to reveal its presence. Multiple imaging modalities should be utilized for tumor localization when androgen-secreting ovarian tumors are suspected on clinical and biochemical grounds to facilitate subsequent treatment.

The patient had a number of features which were compatible with PCOS with its associated metabolic disturbances, including the presence of PCO morphology on ultrasound, diabetes mellitus, overweight and slightly high LH : FSH ratio of 2 (LH: 10.0 IU/L, FSH: 5.0 IU/L). Nevertheless, the presence of clitoromegaly, deepening of voice and the significantly elevated serum 17-OHP and androgen levels prompted us to consider alternative diagnoses. The presence of polycystic ovaries has been reported in a patient with an androgen-secreting SCT–NOS [11] and an androgen-secreting Sertoli–Leydig cell tumor [12]. In both cases, there was enlargement of the involved ovaries leading to gross asymmetry of bilateral ovarian sizes, similar to our case. PCO morphology on ultrasound is a nonspecific finding representing an arrest of follicle development in the antral follicle stage that could be secondary to hyperinsulinemia and/or hyperandrogenism [13, 14]. Apart from hyperandrogenism, our patient also had clinical evidence of insulin resistance (overweight and diabetes mellitus), and both factors might have contributed to the development of the PCO morphology. On the other hand, PCO morphology could be seen in ovulating normal women without evidence of PCOS and may be inconsequential [15, 16]. It is uncertain whether the persistence of the PCO morphology in the left ovary of our patient postoperatively belonged to a part of the spectrum of normal ovulating women or was associated with ongoing insulin resistance. The possibility of coexisting PCOS in this patient was unlikely in view of the resolution of hyperandrogenism and return of normal menstruation after removal of the ovarian tumor.

Steroid cell tumors (SCTs), also known as lipoid cell tumors, are rare ovarian tumors presumably of stromal cell origin and account for 0.1% of ovarian neoplasms [17]. SCTs are classified as Leydig cell tumors when cytoplasmic crystals of Reinke are identified, or when they have a hilar location, nuclear clustering, fibrinoid necrosis of vessels, and have associated hilus cell hyperplasia. They are classified as steroid cell tumor, not otherwise specified (SCT–NOS) when lacking the above features. About 80% of SCTs fall into the NOS category [18]. The average age of diagnosis of SCT–NOS was 43 years. Steroid cell tumors are solid and can range from yellow, orange, red to brown or black. Histologically, these tumors are composed of medium to large polygonal cells with abundant granular eosinophilic (lipid-poor) to pale vacuolated (lipid-rich) cytoplasm, round nuclei, prominent central nucleolus, and variable amounts of intracytoplasmic lipochrome pigment. Nuclear atypia and mitotic activity are rare. The cells are most commonly arranged in diffuse sheets, but can also be seen in nests and cords. The stroma is usually scant and can be oedematous, myxoid, or fibromatous if present. Occasional calcification, necrosis, and haemorrhage can be seen. About one-third of SCT–NOS exhibit malignant behaviour. Predictive features of malignant behaviour include size > 7 cm, > 2 mitoses/ 10 high-power fields (HPFs), necrosis, haemorrhage, and significant nuclear atypia [19]. For immunohistochemistry, steroid cell tumors are positive for sex cord–stromal markers including inhibin, calretinin, steroidogenic factor-1, and CD99. They are also usually positive for Melan-A and negative for S100 and FOXL2 [20]. Around half of SCT–NOS are androgen-secreting [19]. Less commonly, cosecretion of androgens, estradiol, and/or cortisol can occur [21–27].

We reviewed the literature for the association of high 17-OHP levels and SCT–NOS. A PubMed search for cases of SCT–NOS in English using the search string (“steroid cell tumor” or “steroid cell tumour” or “lipoid cell tumor” or “lipoid cell tumor”) was performed. A review of full texts for SCT–NOS with pre-treatment 17-OHP results identified a total of 21 such cases. One case was excluded from data analysis as it was associated with underlying poorly controlled NCCAH due to 21-hydroxylase deficiency [28] (see Table 3 for a summary and Supplementary [Supplementary-material supplementary-material-1] for details). Including our case, all 21 included cases presented with high serum total testosterone concentrations, although the clinical presentation of one case was that of isosexual precocious puberty with mildly elevated serum total testosterone in a 3-year-old girl rather than virilization [24]. Most cases were clinically non malignant (95%). 17 of the 21 cases (81%) presented with elevated 17-OHP levels. Elevation of androstenedione was also common (88%), In contrast, elevation of DHEA-S was uncommon (28%). Furthermore, 7 cases (33%) were associated with hormone cosecretion: 2 cases were associated with cortisol cosecretion [22, 29], 3 cases were associated with estradiol cosecretion [24, 26, 27], and 2 cases were associated with estradiol and cortisol cosecretion [23, 25]. All 7 cases of hormone cosecretion were associated with elevated 17-OHP levels. Among the 17 cases with elevated 17-OHP levels, 5 were initially diagnosed as NCCAH resulting in a delay in diagnosis [23, 26, 30–32]. ACTH stimulation test was performed in 10 cases (including the current case) with elevated baseline 17-OHP levels. No response or insignificant changes were seen in eight cases (80%), including the current case [11, 25, 27, 29, 33–35], while positive 17-OHP responses to ACTH stimulation were observed in two cases (20%) (see legend of Table 3 for the definition of a positive response) [30, 31]. The expression of ACTH receptor may explain the positive 17-OHP response to ACTH stimulation in the minority of cases of SCT–NOS, as the expression of ACTH receptor mRNA was demonstrated in the tumoral tissue of one reported case of SCT–NOS with slightly elevated 17-OHP but not in the ovarian control tissue, although no ACTH stimulation test was performed [24]. Similarly, both 17-OHP responsiveness [36] and nonresponsiveness [37] to ACTH stimulation have been reported in virilizing ovarian Sertoli–Leydig cell tumors. Alternatively, it is possible that the 17-OHP levels in these cases were spuriously increased in response to ACTH stimulation due to cross-reactivity of immunoassays with cortisol and/or other cortisol precursors. Therefore, while a significant increase in 17-OHP in response to ACTH stimulation is typical of CAH [38], this may also be seen in ovarian tumors with 17-OHP hypersecretion, predisposing one to make a wrong diagnosis of CAH in these patients [30, 31].

11-oxopregnanetriol, also known as pregnanetriolone, is a metabolite of 21-deoxycortisol, which is an adrenal-specific metabolite produced by the enzymatic action of 11*β*-hydroxylase on 17-OHP [39, 40]. In both classical and nonclassical forms of 21-hydroxylase deficiency, all three metabolites of 17-OHP (11-oxopregnanetriol, 17-hydroxypregnanolone and pregnanetriol) would be elevated [39, 41, 42]. In contrast, an ovarian source of 17-OHP is associated with the elevation of 17-hydroxypregnanolone and pregnanetriol only [43] (hereinafter referred to as “ovarian pattern”), as exemplified in a report of a 35-year-old woman with an androgen- and 17-OHP-secreting SCT-NOS, in which the 17-OHP concentration was 100.5 nmol/L [44]. It appears that the “ovarian pattern” of 17-OHP metabolism in USP was maintained even at such a high 17-OHP concentration without an “overflowing” of 17-OHP metabolism to the production of 11-oxopregnanetriol. Therefore, obtaining a USP in the investigation of female hyperandrogenism with elevated 17-OHP could be helpful in determining the source of 17-OHP excess (ovarian versus adrenal) and excluding NCCAH. Although unproven, the diagnostic accuracy of USP might theoretically be affected by the ectopic expression of 11*β*-hydroxylase in SCT–NOS, as demonstrated by the presence of its mRNA in one reported case [24]. Therefore, further studies are required to evaluate the diagnostic accuracy of this test in differentiating the origin of 17-OHP hypersecretion.

## 4. Conclusion

In summary, we have described a hormonally active ovarian SCT–NOS in a 24-year-old woman resulting in significant androgen excess, hypersecretion of 17-OHP with a negative response to ACTH stimulation, and an “ovarian pattern” of metabolites of 17-OHP detected in the urine. The case required careful consideration of the clinical, biochemical, and radiological data to clinch the correct diagnosis. A literature review revealed that 17-OHP hypersecretion is commonly seen in SCT–NOS and some patients with SCT–NOS were misdiagnosed as having CAH. Both ACTH stimulation test and USP are helpful biochemical tests to determine the cause of 17-OHP hypersecretion in these patients.

## Figures and Tables

**Table 1:** Pre- and postoperative laboratory results.

Tests (serum/plasma)	Concentration (before operation)	Concentration (2 weeks after operation)	Concentration (4 weeks after operation)	Reference interval
Luteinizing hormone (IU/L)	10.0	4.5	—	2.4–12.6 (follicular phase)
				14.0–95.6 (ovulation phase)
				1.0–11.4 (luteal phase)
Follicle-stimulating hormone (IU/L)	5.0	4.0	—	3.5–12.5 (follicular phase)
				4.7–21.5 (ovulation phase)
				1.7–7.7 (luteal phase)
Estradiol (pmol/L)	161	302	—	98–571 (follicular phase)
				177–1153 (ovulation phase)
				122–1094 (luteal phase)
Progesterone (nmol/L)	—	18.7	—	0.6–4.7 (follicular phase)
				2.4–9.4 (ovulation phase)
				5.3–86 (luteal phase)
Testosterone (nmol/L)	10.6	0.7	0.5	<1.7
Prolactin (mIU/L)	See footnote^a^	See footnote^a^	—	<496
Androstenedione (nmol/L)	28.2	4.2	3.0	1.1–6.5
DHEA-S^b^ (*µ*mol/L)	6.0	6.7	5.9	1.0–11.7
17-OHP (nmol/L)	52	4.3	0.7	0.6–4.0 (follicular phase)
				1.0–6.0 (luteal phase)
Cortisol (1 mg overnight dexamethasone suppression test) (nmol/L)	21	—	—	<50
Fasting glucose (mmol/L)	7.2^c^	6.0	6.5	≥7.0: Diabetes mellitus
HbA1c (%)	6.9^c^	—	—	≥6.5%: Diabetes mellitus
CA125 (kU/L)	17	—	—	<35
Alpha-fetoprotein (*µ*g/L)	8	—	—	<9
Human chorionic gonadotropin (IU/L)	<1	—	—	<1 (premenopausal non pregnant)

^a^Macroprolactin present, value within reference limits after PEG precipitation. ^b^DHEAS: dehydroepiandrosterone-sulphate. ^c^Results obtained at diagnosis, before initiation of anti-diabetic medication.

**Table 2:** ACTH (Synacthen) stimulation test.

	0 min	30 min	60 min	Reference interval
ACTH (pmol/L)	2.4	—	—	<10.2
Cortisol (nmol/L)	239	614	691	—
17-OHP (nmol/L)	46	35	37	—

**Table 3:** A summary of steroid cell tumors, not otherwise specified (SCT–NOS) with 17-OHP concentration reported (*n* = 21, including the current case). Please refer to Supplementary [Supplementary-material supplementary-material-1] for a detailed summary of all cases.

		*n*	Reference
Age (years)^b^	23 (3–68)^a^	21	[9, 11, 22–27, 29–35, 44–48]
Extraovarian (%)	14 (3/21)	21	
Tumor size (cm)^c^	4.9 (1–12)^a^	20	[9, 11, 22, 24–27, 29–35, 44–48]
Evidence of malignancy (%)	5 (1/21)	21	[9, 11, 22–27, 29–35, 44–48]
Serum testosterone concentration (nmol/L)^d^	12 (1.2–37)^a^	21	
Elevated testosterone concentration (%)	100 (21/21)	21	
Serum DHEA-S concentration (*µ*mol/L)^d^	2.9 (0.6–19.7)^a^	14	[9, 11, 23, 24, 26, 27, 29–32, 44, 45, 48]
Elevated DHEA-S concentration (%)	28 (5/18)	18	[9, 11, 23, 24, 26, 27, 29–34, 44–48]
Serum androstenedione concentration (nmol/L)^d^	35 (6.3–78)^a^	15	[9, 23–27, 29, 31–35, 44, 45]
Elevated androstenedione concentration (%)	88 (14/16)	16	[9, 23–27, 29, 31–35, 44, 45, 47]
Serum 17-OHP concentration (nmol/L)^d^	48 (1.8–312)^a^	16	[9, 11, 23–27, 29–32, 35, 44, 45, 48]
Elevated 17-OHP concentration (%)	81 (17/21)	21	[9, 11, 22–27, 29–35, 44–48]
Positive 17-OHP response after 1–24 ACTH stimulation (%)^e^	20 (2/10)	10	[11, 25, 27, 29–31, 33–35]
Hormonal cosecretion (hormones other than androgens) (%)	33 (7/21)	21	[9, 11, 22–27, 29–35, 44–48]
Cortisol	10 (2/21)		
Estradiol	14 (3/21)		
Estradiol and cortisol	10 (2/21)		

^a^Results expressed as median (range). ^b^Age at diagnosis. ^c^Largest dimension. If tumor size was not reported, the size of the affected ovary was used. ^d^For results reported as “larger than X units”, the value of X was used. For cases in which the diagnosis was delayed, the values at the age of diagnosis was used. Cases with no values reported (stated as “normal”), suspected errors in reporting units or unknown conversion factor to SI units were excluded. ^e^Defined as positive if commented to be increased from baseline by authors and peak 17-OHP >30 nmol/L. Otherwise, it was arbitrarily defined an increase in 17-OHP, at either 30 min or 60 min after ACTH stimulation, by equal to or more than 100% of the basal value, and a peak value >30 nmol/L.

## Supplementary Materials

Supplementary Materials Supplementary Table 1: summary of cases of steroid cell tumors, not otherwise specified (SCT–NOS) with 17-OHP concentration reported.
