# Supplementary material for: Hyperandrogenism, Elevated 17-Hydroxyprogesterone and Its Urinary Metabolites in a Young Woman with Ovarian Steroid Cell Tumor, Not Otherwise Specified: Case Report and Review of the Literature
Source: Case Rep Endocrinol. 2019 Oct 27;2019:9237459. doi: 10.1155/2019/9237459 (PMC6854983; doi:10.1155/2019/9237459)
Supplement: Supplementary Materials — Supplementary Table 1: summary of cases of steroid cell tumors, not otherwise specified (SCT–NOS) with 17-OHP concentration reported. [file 9237459.f1.docx]

Supplementary Table 1

Summary of cases of steroid cell tumors, not otherwise specified (SCT-NOS) with 17-OHP concentration reported.

| Reference | Age at symptom onset (years) | Age at diagnosis (years) | Clinical presentation | Tumor size (or size of the affected ovary if tumor size not available) (cm) | Evidence of malignancy? | Serum testosterone concentration (nmol/L) | Serum DHEA-S concentration (μmol/L) | Serum androstenedione concentration (nmol/L) | Serum 17-OHP concentration (nmol/L) | | 17-OHP concentration after 1-24 ACTH stimulation (if performed) (nmol/L)^a^ | Treatment | Improvement/resolution of symptoms after treatment? | Remarks |
| --- | --- | --- | --- | --- | --- | --- | --- | --- | --- | --- | --- | --- | --- | --- |
| **Elevated 17-OHP** | | | | | | | | | | | | | | |
| Adashi, 1979 [1] | 14 | 16 | Secondary amenorrhea, hirsutism, voice deepening, obesity, male type escutcheon, clitoromegaly | 10 x 6 x 7 (tumor) | No | 16 (high) | N/A | 78 (high) | 56 (high) | | 30 min: 59 Commented to be an insignificant rise | Right salpingo-oophrectomy | Yes | -- |
| Imperato-McGinley, 1981 [2] | 4 | 23 | Precocious puberty at age 4. Hirsutism, acne, oligomenorrhea followed by secondary amenorrhea. | 3 x 1 x 1 (tumor) | No | 8.0 (high) | N/A | 49 (high) | 120 (high) | | Before infusion: 105  After infusion: 140  Commented as no increase after IV administration of 40 units of ACTH over 8 hours. | Tumor removal with approximately one-third of normal ovarian tissue left | Yes | The tumor was androgen-, cortisol- and estradiol-secreting |
| Chetkowski, 1985 [3] | 15 | 20 | Hirsutism, acne, amenorrhea, male escutcheon | 5 x 6 x 3 (tumor) | No | 5.2 (high) | 2.4 (normal) | 32 (high) | 312 (high) | | Before: 329  60 min: 313  Commented as no response | Right salpingo-oophorectomy | Yes | The tumor was androgen- and cortisol-secreting. |
| Harris, 1991 [4] | 6 | 8 | Accelerated growth, heterosexual precocity, virilization, initially treated as NCCAH | 3.5 x 3.5 x 2 (tumor) | No | 25.7 (high) | 2.1 (slightly high) | 76.1 (high) | 60.4 (high) | | N/A | Left oophorectomy | Yes, with persistent deep voice | -- |
| Azizlerli, 1997 [5] | 8 | 21 | Accelerated linear growth and virilisation, previously treated as NCCAH | 6 x 3.5 x 1.5 (tumor) | No | At the age of 8 years: 39.1 (high)  At the age of 21 years  16 (high) | At the age of 21 years: 5.6 (no reference interval given, likely normal) | At the age of 21 years: 41 (no reference interval given, likely high) | At the age of 8 years:  >60.5 nmol/L (high)  At the age of 21 years:  257 nmol/L (high) | | N/A | Right oophorectomy | Yes, with persistent clitoromegaly | The tumor was androgen- and estradiol-secreting. |
| Lin, 2000 [6] | 2 | 3 | Isosexusal precocious puberty, accelerated growth | 4.4 x 4.1 x 3.2 (tumor) | No | 1.2 (slightly high) | 3.1 (high) | 6.3 (high) | 3.9 (slightly high) | | N/A | Right oophorectomy | Yes | The tumor was androgen- and estradiol-secreting |
| Dumic， 2001 [7] | 7 | 12 | Premature pubarche, hirsutism, acne, voice deepening, rapid increase in height, increased muscle mass and clitoromegaly. Initally treated as NCCAH | 5 x 4 x 3 (tumor) | No | 6.3 – 17.7 (high) | 2.2 – 3.1 (normal) | 14.3 – 44.0 (high) | 14.1 – 49.1 (high) | | 60 min: 44.9  Commented as increased from baseline, though exact baseline value unknown. | Complete tumor extirpation | Yes | Tumor located at the infundibulopelvic ligament, i.e. extraovarian  Diagnosis of CAH excluded by DNA analysis |
| Smith，2006 [8] | 5 | 11 | Premature pubarche, acne, increased muscle bulk, frontal temporal recession of scalp hair, clitoromegaly | 2 (tumor) | No | 8.8 (high) | 4.1^b^ (normal) | 34.5 (high) | 21.1 mU/L^c^ (high) | | Commented as no response (concentration not reported) | Cyproterone, nafereline, then tumor resection | Yes | Tumor arising within the left broad ligament, i.e. extraovarian. |
| Stephens, 2008 [9] | 34 | 35 | Hirsutism, secondary amenorrhea with preceding oligomenorrhea, increased muscle mass, voice deepening | 5 x 5 x 3 (tumor) | No | 28.3 (high) | 19.7 (high) | >34.7 (high) | 100.5 (high) | | N/A | Right salpingo-oophorectomy, wedge resection of the left ovary | Yes | Right ovarian tumor.  USP was performed. |
| Gupta， 2008 [10] | 2 | 5 | Premature pubarche diagnosed as NCCAH at age of 2 with lost to follow up.  Presented again at age 5 with Cushingoid features, abdominal mass, proximal muscle weakness, hirsutism, acne advanced growth, clitoromegaly and generalized hypertrichosis | N/A | No | At 2 years: 5.5 (high)  At 5 years: 9.2 (high) | At 2 years: 0.8 (normal)  At 5 years: 2.8 (high) | At 5 years: 57 (high) | At 2 years: 43 (high)  At 5 years: 44 (high) | | N/A | Excision of the ovarian mass | Yes | The tumor was estradiol-, androgen- and cortisol-secreting. |
| Yılmaz-Ağladıoğlu, 2013 [11] | 6 | 13 | Premature pubarche, diagnosed with NCCAH by another centre at the age of 6 years  At 13 years of age, hirsutism, increased muscle mass, clitoromegaly and voice deepening | 2.5 x 2.3 x 2.1 (tumor) | No | At the age of 13: 5.1 (RI not provided, likely high) | At the age of 13: 3.0  (RI not provided, likely normal) | N/A | | At the age of 6:  18 (high)  At the age of 13:  58 | At the age of 6:  30 min: 30  60 min: 51 | Tumor resection sparing the right ovary | Yes, with persistent deep voice, menarche 6 months after surgery | NCCAH excluded by postoperative ACTH stimulation test (Peak stimulated 17-OHP was 13 nmol/L) |
| Thomas, 2013 [12] | Not applicable as the tumor was discovered incidentally | 17 | Underlying CAH due to 21-hydroxylase deficiency diagnosed at birth. Incidental finding of left ovarian mass on ultrasound screening. | 5 (tumor) | No | >1.7 (high) | N/A | N/A | >151 (high) | | N/A | Left oophorectomy | Not applicable as the tumor was discovered incidentally | Excluded from data analysis as the patient has underlying NCCAH due to 21-hydroxylase deficiency. |
| Lambrinoudaki, 2015 [13] | 65 | 67 | Postmenopausal hirsutism and hair loss | 3.0 x 2.4 x 2.4 (ovary) | No | 12 (high) | 2.6^d^ (normal) | 14.3 (high) | | 28^e^ (high) | 35^e^  Commented to be a normal response | Triptorelin, then bilateral oophorectomy and hysterectomy | Yes | -- |
| Lee， 2016 [14] | 42 | 47 | Male pattern alopecia, hirsutism, amenorrhea. | 11 x 8 x 4 (tumor) | Yes, with pelvic cul-de-sac metasis (4.9 cm x 4 cm) | 12 (high) | 0.6 (normal) | 57 (high) | 18 (high) | | N/A | Total abdominal hysterectomy,  bilateral salpingo-oophorectomy, omentectomy, and resection of cul-de-sac tumor with no gross residual disease | Yes. Alive and free of disease 24 months after surgery. | -- |
| Zang， 2017 [15] | 45 | 46 | Secondary amenorrhea, hirsutism, clitoromegaly, facial plethora, hypertension, hypokalemia (3.41 mmol/L) | 12 x 8 (tumor) | No | 37 (high) | N/A | N/A | 7.1^f^ (high) | | N/A | Left adnexectomy | Yes | The tumor arised from an accessory ovary, i.e. extraovarian. It was androgen- and cortisol-secreting. |
| Benavent Correro, 2018 [16] | 64 | 68 | Androgenic alopecia without hirsutism or acne. Increased muscle mass, clitoromegaly | 4.8 (tumor) | No | 16 (high) | 6.1 (high) | >35 (high) | 27.6 (high) | | 30 min: 29.4 | Bilateral salpingo-oophorectomy and hysterectomy | Normalization of steroid concentrations. Symptomatic improvement not mentioned. | The tumor was androgen- and estradiol-secreting. |
| Kale, 2019 [17] | 22.5 | 23 | 6 months duration of hirsutism, male pattern baldness, clitoromegaly, oligomenorrhea followed by amenorrhea | 3.6 x 3.2 x 2.6 (tumor) | No | 24 (high) | 8.5 (normal) | N/A | 13 (high) | | 60 min: 26 | Right salpingo-oophorectomy | Yes | -- |
| Wong, 2019 (Current case) | 22 | 24 | Secondary amenorrhea, hirsutism, voice deepening, clitoromegaly | 4.5 x 4.5 (ovary) | No | 10.6 (high) | 6.0 (normal) | 28.2 (high) | 52 (high) | | Baseline: 46  30 min: 35  60 min: 37 | Laparoscopic right salpingo-oophorectomy | Yes | -- |
| **Normal 17-OHP** | | | | | | | | | | | | | | |
| Cserepes, 2002 [18] | 47 | 49 | Hirsutism, voice deepening, male pattern hair recession | 1 (tumor) | No | 14 (high) | 2.2 (low) | 8.4 (normal) | 2.6 (normal) | | N/A | Bilateral salpingo-oophorectomy | Yes | A 1.5 cm thecoma was also identified in the left ovary. Preoperative selective venous sampling localized the hormonal secretion to the right side |
| Varras, 2011 [19] | 37 | 40 | Hirsutism, temporal and parietal hair recession, overdeveloped muscles, clitoromegaly | 6.8 x 5.5 x 3.5 (tumor) | No | 8.8 (high) | 0.27^g^ (normal) | N/A | Normal (concentration not reported) | | N/A | Total hysterectomy and bilateral salpingo-oophorectomy | Yes | -- |
| Boyraz, 2013 [20] | 15.5 | 16 | 6 months history of hirsutism, acne, amenorrhea | 6 × 4 × 3.3 (tumor) | No | 3.3 (minimally elevated) | Normal (concentration not reported) | Normal (concentration not reported) | Normal (concentration not reported) | | N/A | Right ovarian cystectomy | Yes | -- |
| Swain, 2013 [21] | 26 | 28 | Lactation failure, hirsutism, amenorrhea, mild intermittent abdominal pain, male body habitus, temporal recession of hairline, clitoromegaly, atrophy of bilateral mammary gland | 5.2 x 5.9 x 5.9 (tumor) | No | 24 (high) | 2.2 (normal) | N/A | 1.8 (normal) | | N/A | Left salpingo-oophorectomy | Yes | -- |

All results were converted to SI units unless otherwise stated. N/A: not available. ^a^ Refer to the column to the left (“Serum 17-OHP concentration (nmol/L)”) for the baseline result if not otherwise stated. ^b^ Result reported as 4.1 nmol/L. An error in the unit was suspected. A correct unit of μmol/L was assumed. ^c^ Unknown conversion factor to SI unit. ^d^ Result reported as 95 mg/dl. An error in the unit was suspected. A correct unit of µg/dL was assumed for conversion to SI unit. ^e^ Results reported as 9.3 ng/dl and 11.6 ng/dl before and after ACTH stimulation, respectively. An error in the unit was suspected. A correct unit of ng/ml was assumed for conversion to SI unit. ^f^ Result reported as 2.347 ng/dL. An error in the unit was suspected. A correct unit of ng/ml was assumed for conversion to SI unit. ^g^ Result reported as 10 mg/dl. An error in the unit was suspected. A correct unit of µg/dL was assumed for conversion to SI unit.

Results with probable erroneous units (b, d-g) or unknown conversion to SI unit (c) were excluded from the generation of descriptive statistics (median and range, see Table 3 in main text).

Reference

1 Adashi EY, Rosenwaks Z, Lee PA, Jones GS, Migeon CJ: Endocrine features of an adrenal-like tumor of the ovary. J Clin Endocrinol Metab 1979;48:241–245.

2 Imperato-McGinley J, Peterson RE, Dawood MY, Zullo M, Kramer E, Saxena BB, et al.: Steroid hormone secretion from a virilizing lipoid cell tumor of the ovary. Obstet Gynecol 1981;57:525–531.

3 Chetkowski RJ, Judd HL, Jagger PI, Nieberg RK, Chang RJ: Autonomous cortisol secretion by a lipoid cell tumor of the ovary. JAMA 1985;254:2628–2631.

4 Harris AC, Wakely PE, Kaplowitz PB, Lovinger RD: Steroid cell tumor of the ovary in a child. Arch Pathol Lab Med 1991;115:150–154.

5 Azizlerli H, Tanakol R, Terzioğlu T, Alagöl F, Dizdaroglu F: Steroid cell tumor of the ovary as a rare cause of virilization. Mt Sinai J Med 1997;64:130–135.

6 Lin CJ, Jorge AA, Latronico AC, Marui S, Fragoso MC, Martin RM, et al.: Origin of an ovarian steroid cell tumor causing isosexual pseudoprecocious puberty demonstrated by the expression of adrenal steroidogenic enzymes and adrenocorticotropin receptor. J Clin Endocrinol Metab 2000;85:1211–1214.

7 Dumic M, Simunic V, Ilic-Forko J, Cvitanovic M, Plavsic V, Janjanin N, et al.: Extraovarian steroid cell tumor “not otherwise specified” as a rare cause of virilization in twelve-year-old girl. Horm Res 2001;55:254–257.

8 Smith D, Crotty TB, Murphy JF, Crofton ME, Franks S, McKenna TJ: A steroid cell tumor outside the ovary is a rare cause of virilization. Fertil Steril 2006;85:227.

9 Stephens JW, Fielding A, Verdaguer R, Freites O: A steroid-cell tumor of the ovary resulting in massive androgen excess early in the gonadol steroidogenic pathway. Gynecol Endocrinol 2008;24:151–153.

10 Gupta P, Goyal S, Gonzalez-Mendoza LE, Noviski N, Vezmar M, Brathwaite CD, et al.: Corticotropin-independent cushing syndrome in a child with an ovarian tumor misdiagnosed as nonclassic congenital adrenal hyperplasia. Endocr Pract 2008;14:875–879.

11 Yılmaz-Ağladıoğlu S, Savaş-Erdeve Ş, Boduroğlu E, Önder A, Karaman İ, Çetinkaya S, et al.: A girl with steroid cell ovarian tumor misdiagnosed as non-classical congenital adrenal hyperplasia. Turk J Pediatr 2013;55:443–446.

12 Thomas TT, Ruscher KR, Mandavilli S, Balarezo F, Finck CM: Ovarian steroid cell tumor, not otherwise specified, associated with congenital adrenal hyperplasia: rare tumors of an endocrine disease. J Pediatr Surg 2013;48:E23-27.

13 Lambrinoudaki I, Dafnios N, Kondi-Pafiti A, Triantafyllou N, Karopoulou E, Papageorgiou A, et al.: A case of postmenopausal androgen excess. Gynecol Endocrinol 2015;31:760–764.

14 Lee J, John VS, Liang SX, D’Agostino CA, Menzin AW: Metastatic Malignant Ovarian Steroid Cell Tumor: A Case Report and Review of the Literature. Case Rep Obstet Gynecol 2016;2016. DOI: 10.1155/2016/6184573

15 Zang L, Ye M, Yang G, Li J, Liu M, Du J, et al.: Accessory ovarian steroid cell tumor producing testosterone and cortisol. Medicine (Baltimore) 2017;96. DOI: 10.1097/MD.0000000000007998

16 Benavent Correro P, Sáenz Valls M, García Cano A, Jiménez Mendiguchia L, Moreno Moreno E, Luque-Ramírez M: An unusual circulating steroid profile in a virilized postmenopausal woman. Diagnosis (Berl) 2018;5:83–87.

17 Kale K, Chauhan AR, Kalappa S: Virilization Secondary to Androgen-Secreting Tumor of the Ovary: A Report of Three Cases and Review of Literature. J Obstet Gynaecol India 2019;69:56–59.

18 Cserepes E, Szücs N, Patkós P, Csapó Z, Molnár F, Tóth M, et al.: Ovarian steroid cell tumor and a contralateral ovarian thecoma in a postmenopausal woman with severe hyperandrogenism. Gynecol Endocrinol 2002;16:213–216.

19 Varras M, Vasilakaki T, Skafida E, Akrivis C: Clinical, ultrasonographic, computed tomography and histopathological manifestations of ovarian steroid cell tumour, not otherwise specified: our experience of a rare case with female virilisation and review of the literature. Gynecol Endocrinol 2011;27:412–418.

20 Boyraz G, Selcuk I, Yusifli Z, Usubutun A, Gunalp S: Steroid Cell Tumor of the Ovary in an Adolescent: A Rare Case Report. Case Reports in Medicine 2013; DOI: 10.1155/2013/527698

21 Swain J, Sharma S, Prakash V, Agrawal NK, Singh SK: Steroid cell tumor: a rare cause of hirsutism in a female. Endocrinol Diabetes Metab Case Rep 2013;2013. DOI: 10.1530/EDM-13-0030
